# Control of Bleeding in Endoscopic Skull Base Surgery: Current Concepts to Improve Hemostasis

**DOI:** 10.1155/2013/191543

**Published:** 2013-06-13

**Authors:** Cattleya Thongrong, Pornthep Kasemsiri, Ricardo L. Carrau, Sergio D. Bergese

**Affiliations:** ^1^Department of Anesthesiology, Wexner Medical Center at The Ohio State University, Columbus, OH, USA; ^2^Department of Anesthesiology, Srinagarind Hospital, Faculty of Medicine, 123 Mitraparp Highways, Khon Kaen University, Khon Kaen 40002, Thailand; ^3^Department of Otolaryngology, Head and Neck Surgery, Wexner Medical Center at The Ohio State University, Columbus, OH, USA; ^4^Department of Otorhinolaryngology, Srinagarind Hospital, Faculty of Medicine, Khon Kaen University, Khon Kaen, Thailand; ^5^Department of Neurological Surgery, Wexner Medical Center at The Ohio State University, Columbus, OH, USA

## Abstract

Hemostasis is critical for adequate anatomical visualization during endoscopic endonasal skull base surgery. Reduction of intraoperative bleeding should be considered during the treatment planning and continued throughout the perioperative period. Preoperative preparations include the optimization of comorbidities and cessation of drugs that may inhibit coagulation. Intraoperative considerations comprise anesthetic and surgical aspects. Controlled hypotension is the main anesthetic technique to reduce bleeding; however, there is controversy regarding its effectiveness; what the appropriate mean arterial pressure is and how to maintain it. In extradural cases, we advocate a mean arterial pressure of 65–70 mm Hg to reduce bleeding while preventing ischemic complications. For dealing intradural lesion, controlled hypotension should be cautious. We do not advocate a marked blood pressure reduction, as this often affects the perfusion of neural structures. Further reduction could lead to stroke or loss of cranial nerve function. From the surgical perspective, there are novel technologies and techniques that reduce bleeding, thus, improving the visualization of the surgical field.

## 1. Introduction

 Endoscopic surgery is a minimally invasive technique that has found a niche in all surgical fields. Endoscopic endonasal surgery ranges from basic and relatively straightforward procedures (e.g., endoscopic septoplasty, endoscopic turbinoplasty, and functional endoscopic sinus surgery) to advanced surgery (e.g., endoscopic orbital and/or optic nerve decompression, endoscopic dacryocystorhinostomy, and endoscopic endonasal skull base approaches). Its advantages are obviating external scars, reducing damage to normal tissue and bone, and shortening recovery time and length of hospital stay. However, intraoperative bleeding presents a larger obstacle to endoscopic visualization. Blood obscures the anatomy of the surgical field and dirties the endoscope lens causing greater difficulty with visualization. This situation increases the risk of complications, including brain injury, orbital or optic nerve injury, and catastrophic bleeding from major vessels (e.g., internal carotid artery).

We advocate careful consideration of all factors regarding the control of bleeding throughout the entire perioperative period. Preoperative preparations include the optimization of co-morbidities and cessation of drugs that may increase the tendency for bleeding. Intraoperative considerations comprise anesthetic and surgical aspects. Some anesthetic aspects are controversial including the use of controlled hypotension and whether inhalation-based or intravenous-based technique is most effective in reducing intraoperative bleeding. Additionally, novel surgical technologies, materials, and techniques help to improve the quality of surgical visualization. This paper aims to review controversies and current concepts regarding how to minimize intraoperative bleeding in endoscopic endonasal skull base surgery. We classify endoscopic endonasal skull base surgery into extradural and intradural surgery. The principles of extradural surgery are similar to those of the endoscopic sinus surgery.

## 2. Preoperative Evaluation

### 2.1. Dealing with Bleeding Disorders

Coagulation comprises three major components: vascular compartment, platelets, and coagulation factors [1]. Inherited and acquired coagulopathies can arise from any of these components. Vascular problems include hereditary hemorrhagic telangiectasia (e.g., Osler-Weber-Rendu syndrome), which is an inherited disorder commonly occurring on the face and nose. Platelet-related bleeding disorders can occur by a low number of available platelets (thrombocytopenia), by dysfunction of their aggregation, or by a combination of the two (e.g. idiopathic thrombocytopenic purpura, renal failure, liver failure, medications such as aspirin, nonsteroidal anti-inflammatory drugs [NSAIDs], and dietary supplements such as vitamin E, fish oil, *Echinacea, Aloe,* and garlic extract). Disorders related to deficiencies of the coagulation factors include hemophilia, von Willebrand's disease, and medications such as warfarin and heparin (Table 1).

Anamnesis should include symptoms specific to bleeding, such as a history of unexplained bleeding associated with surgical procedures, trauma, or menses, unexplained bruises or hematomas family history suggesting bleeding tendency and use of prescribed and over-the-counter medications and supplements. However, patients may be asymptomatic; therefore, physical examination should investigate the presence of petechiae, ecchymotic lesions, telangiectasias, hepato-splenomegaly, and hemarthrosis of the joints. Furthermore, preoperative laboratory evaluations, including platelet count, prothrombin time, and activated prothrombrin time, are helpful to confirm a suspected diagnosis and for the preoperative planning. However, systematic screening is inefficient and should be confined to patients at high risk [2]. Bleeding time is purported to evaluate platelet function, but it is associated with poor accuracy due to operator technique and patient variation (e.g., skin thickness, skin temperature, and vascular pattern) [3, 4]. Therefore, this test is not recommended [5].

Identification and preoperative management of patients who are receiving anticoagulants and/or antiplatelet medication cannot be emphasized. Current recommendations include holding both aspirin and clopidogrel for 7–10 days prior to surgery to allow new platelets to replace those that are dysfunctional [6]. Warfarin is usually discontinued 5 days prior to surgery, which is an adequate time span to reconstitute the coagulation factors; however, this depends on the condition of each patient. Recent studies demonstrated that most procedures can be done if the international normalized ratio (INR) is less than 1.5 [6, 7]. Another study demonstrated that most patients had an INR of less than 1.5 after discontinuing warfarin for 5 days [8]. If the patient is at moderate to high risk for thromboembolic phenomena, the recommendation is to use low-molecular-weight heparin (LMWH) to “bridge” the effect of the two anticoagulant therapies. One should start the LMWH three days before surgery or two days after discontinuing warfarin, stopping it approximately 24 hours prior to surgery [9].

### 2.2. Preoperative Embolization

Vascular tumors present a surgical challenge. Preoperative embolization of vascular tumors reduces bleeding, and the need for transfusion, as well as improves the visualization of intraoperative surgical field [10]. Embolization should be performed 24 to 72 hours before surgery to allow adequate thrombosis while avoiding recanalization and/or formation of collateral blood supply. Embolic agents include polyvinyl alcohol, transacryl microspheres, liquid n-butylcyanoacrylate (n-BCA), ethyl-vinyl alcohol copolymer (EVOH), and microcoils [11]. These agents occlude the main arterial feeder and may advance further downstream to occlude the blood supply at the capillary level. Traditional transarterial embolization is the most common technique; however, reports of alternative direct tumor puncture show some promise claiming greater efficacy. A claim of greater safety, seemingly due to lower risk for the migration of agents through nontarget arterial vessels, should be taken in context. An initial experience with this technique was fraught with catastrophic complications including blindness, stroke, and death [12, 13]. 

### 2.3. Preoperative Corticosteroid Administrations

 Inflammatory mediators produce diffuse vasodilatation, transudation, and edema in the sinonasal mucosa. Thus, preoperative administration of steroids is beneficial due to their anti-inflammatory and antiedematous effects. These advantages include having a wider nasal corridor, thus, improving visibility of the surgical field. A recent study demonstrated that corticosteroids significantly improved the hemostasis within the operative field (*P* = 0.03) and shortened the surgery time (*P* = 0.041) in patients with chronic polypoid rhinosinusitis [14].

## 3. Anesthesia Considerations

### 3.1. Controlled Hypotension

Controlled hypotension technique includes various modalities associated with different potency and adverse effects. Its safety is dependent on a thorough knowledge about the mechanism of action for each modality, adequate monitoring of the patient, and choosing the appropriate modality with consideration to history of drug allergies and co-morbidities.

#### 3.1.1. Patient's Positioning

Positioning is important for the safety of the patient and to facilitate the surgeon's ergonomic approach. Reverse Trendelenburg or anti-Trendelenburg position is a common surgical position in which the head is up and feet are down. Head elevation reduces mean arterial pressure in the elevated part by about 2 mm Hg for each 2.5 cm above the cardiac level [15]. A reverse Trendelenburg position is used in numerous surgical procedures and presents multiple benefits. It reduces venous return from the lower extremities, therefore, reducing total blood loss (*P* < 0.001), blood loss per minute (*P* < 0.001) and improving hemostasis of the surgical field (*P* = 0.004) when compared with the supine position [16]. Sudden shift in blood pressure is a serious complication of the reverse Trendelenburg position; the patient must be tilted in and out slowly to avoid this complication.

#### 3.1.2. Using a Laryngeal Mask Airway

The laryngeal mask airway (LMA) is a supraglottic device that is associated with less respiratory and cardiovascular reflex responses due to reduced stimulation of the larynx as compared to endotracheal intubation. Moreover, LMA facilitates controlled hypotension. One study suggested that LMA is more effective than endotracheal intubation in regard to rapid onset to achieve a target systolic arterial blood pressure (*P* < 0.05), less blood loss (200 ± 96.8 mL; *P* < 0.05), and use of lower doses of remifentanil (*P* < 0.05). Visibility of the operative field improved in the first 15 minutes (*P* < 0.05) [17].

#### 3.1.3. Technique of Ventilation

Ventilation with normocapnia or mild hypocapnia has been advocated to minimize bleeding and optimize the surgical field during endoscopic endonasal skull base surgery. However, a recent study demonstrated no significant difference in the surgical conditions or blood loss among hypocapnia (end tidal carbon dioxide (ETCO_2_)  27 ± 2 mm Hg), normocapnia (ETCO_2_  37 ± 2 mm Hg), or hypercapnia (ETCO_2_  60 ± 2 mm Hg) in patients under propofol plus remifentanil anesthesia when heart rate and blood pressure were controlled [18]. Although this study showed that hypercapnia does not yield significantly different intraoperative bleeding, it is known that high hypercapnia produces vasodilatation, increasing cerebral blood flow and brain fullness. These conditions are detrimental to endoscopic approaches to the skull base.

Furthermore, mode of ventilation is important to control hypotension. Traditional intermittent positive pressure ventilation (IPPV) has a troublesome hemodynamic effect due to high intrathoracic pressures and reduced venous return to the heart [19]. This result provides decreased blood circulation from the upper part of the body; therefore, it is a high risk for intraoperative bleeding. Conversely, high-frequency jet ventilation (HFJV) is small volume ventilation that provides adequate gas exchange at a lower pressure than intermittent positive pressure ventilation. A recent study compared efficacy between intermittent positive pressure ventilation and high frequency jet ventilation demonstrating that the total mean blood loss in the high-frequency jet ventilation group (170 mL) was significantly lower than the intermittent positive pressure ventilation group (318.8 mL; *P* = 0.017). The quality of the surgical field in high-frequency jet ventilation was significantly better than the intermittent positive pressure ventilation group (*P* = 0.012) [20]. However, high-frequency jet ventilation is not practical for skull base surgery.

#### 3.1.4. Medications for Controlled Hypotension

*(1) Inhalation Anesthetics.* Controlled hypotension with inhalation agents (e.g., isoflurane, sevoflurane, and desflurane) decreases arterial blood pressure through peripheral vasodilatation due to blockage of *α*-adrenoceptors. However, higher concentrations can increase cerebral blood flow, increase the intracranial pressure, and deteriorate cerebral autoregulation. Therefore, a combination of inhalation anesthetics with other drugs has been advocated to help reduce the concentration and adverse effects of each agent [21]. Combination of an inhalation agent <1 minimum alveolar concentration (MAC) with other adjuvant drugs prevents adverse events including rebound hypertension, reflex tachycardia, and distressed myocardial function.

*(2) Intravenous Anesthesia.* Intravenous anesthesia has been introduced for analgesia, hypnosis, sedation, and general anesthesia (induction phase or maintenance phases). Agents currently used for general anesthesia include propofol and opioids.

Propofol has a depressant effect on the central nervous system via direct activation of the gamma-aminobutyric acid (GABA [A]) receptors, inhibition of the n-methyl d-aspartate (NMDA) receptor, and modulation of the calcium influx through slow calcium ion channels. Propofol has rapid onset of action and recovery time with a dose-related effect. However, dose-dependent hypotension is its most common complication, especially high-dose infusions that are associated with propofol infusion syndrome. This condition is a potentially fatal complication with severe metabolic acidosis and circulatory collapse [22].

Traditional opioids have been used as analgesic drugs and they bring some hypotensive effect. However, this effect is difficult to use for controlled hypotension due to their long half-life. Remifentanil is a new potent ultrashort-acting *μ* opioid agonist with a short half-life; therefore, its action has a rapid onset and offset. It offers a reduction in sympathetic nervous system tone and dose-dependent effects, decreasing heart rate and blood pressure. 

In general anesthesia, opioids are often used as an adjunct of intravenous-based technique (opioids combined with antihypertensive drugs or propofol) or inhalation-based technique (opioids combined with an inhalation agent). There is controversy regarding whether intravenous- or inhalation-based technique is more efficient for controlled hypotension (Table 2).

*(3) Antihypertensive Drugs.* Antihypertensive drugs have numerous classifications and mechanisms of action to control blood pressure [31]. Controversy exists regarding which is the ideal mean arterial pressure (MAP) in controlled hypotension to reduce bleeding and the correlation between blood loss and MAP (Table 3). MAP at a very low level did not correlate with decreased intraoperative blood loss. However, severe hypotension may further reduced blood supply to vital organs.

Our recommendation allows to maintain MAP at 65–70 mm Hg, to avoid organ hypoperfusion [24, 36]. Although controlled hypotension seems to be effective in reducing intraoperative bleeding during endoscopic sinus surgery, its use during endoscopic endonasal skull base surgery for intradural lesion is cautious. Impairments of blood flow and oxygen supply to the brain and cranial nerves are major considerations. Hugosson and Högström [37] reported one cerebral hypoxia resulting from the induced deep hypotension led to death in their patients who underwent intracranial surgery.

## 4. Surgical Considerations

There are many methods to deal with intraoperative bleeding during endoscopic endonasal skull base surgery. Their choice often depends on whether the bleeding is from venous or arterial origin, the size of the vessel, and its location [38] (Table 4).

### 4.1. Topical Vasoconstrictors

The aim of topical vasoconstrictors is to decongest the nasal cavity, thus widening the nasal corridor and minimizing bleeding. Commonly used topical vasoconstrictors include cocaine, epinephrine, phenylephrine, and oxymetazoline. All topical vasoconstrictors have potential adverse effects; therefore, the property of each agent should be considered.

### 4.2. Local Anesthetic with Vasoconstrictor Injection

Infiltration of a solution of local anesthetic with vasoconstrictor has been introduced to minimize intraoperative bleeding. Hemostatic efficacy of local anesthetic with vasoconstrictor was demonstrated in a study that showed decreased bleeding when lidocaine/epinephrine was injected as compared to injection of placebo (*P* < 0.05) [39]. However, intraoperative estimated blood loss was not significantly different between the anesthetic/epinephrine and control groups (*P* > 0.05) [29, 30]. Furthermore, epinephrine can be injected directly into pterygopalatine fossa (containing the main nasal arterial supply) resulting in significantly better hemostasis (*P* = 0.01) [40].

Arterial pressure and heart rate were affected immediately after injection of lidocaine/epinephrine but were not elevated over the normal range [39]. Another study found no significant difference between bupivacaine/epinephrine and control groups (*P* > 0.05) [41].

### 4.3. Hemostatic Biomaterials

Low-flow bleeding (capillary, venous, and small arteries) can be inhibited by the topical application of absorbable biomaterials. Recent development of numerous biomaterials has provided new methods for effective intraoperative and postoperative hemostasis, while avoiding complications, such as adhesions, excessive granulation tissue, and crusting.

#### 4.3.1. Topical Antifibrinolytics

Topical antifibrinolytics (i.e., epsilon-aminocaproic acid, tranexamic acid) mechanism of action is competitive binding with the lysine site on plasminogen. This prevents fibrinolysis and stabilizes the blood clot potentially decreasing further bleeding. However, the epsilon-aminocaproic acid was demonstrated to be ineffective in reducing intraoperative bleeding. Conversely, a low dose (100 mg) of tranexamic acid provided hemostasis and improved quality of the surgical field at 2, 4, and 6 minutes after application (*P* < 0.05). No adverse effects were found after topical application [42].

#### 4.3.2. Gelatin-Thrombin Matrix

Topical matrix sealant consists of human thrombin and gelatin matrix granules of bovine or porcine gelatin. It provides tamponade of injured vessels and rapid clot formation on the tissue surface. Topical gelatin-thrombin matrixes have been modified to allow their use during endoscopic skull base endonasal surgery. It stops bleeding on an average of 2 minutes (range 1–5 minutes) after its application [43]. In addition, a recent study showed that bovine gelatin can effectively stop bleeding from the venous sinus [44, 45]. Some studies reported its adverse effects, including extensive loss of cilia on the epithelium [46], significant increase of adhesion (*P* < 0.05) and of granulation tissue formation (*P* < 0.05) [47, 48]. 

A prospective one-arm study evaluated the efficacy and complications associated with porcine gelatin. Hemostasis was achieved within 10 minutes after application. No serious adverse effects, such as synechiae, adhesions, or infections, were encountered [49].

#### 4.3.3. Microporous Polysaccharide Hemispheres

Microporous polysaccharide hemispheres are a novel hemostatic biomaterial agent produced from purified potato starch that acts to dehydrate blood and concentrate bloody components, including platelets, red blood cells, and clotting factors. One study demonstrated its hemostatic effect at approximately 30 to 45 seconds and no significant break-through bleeding in 5 minutes after application [50]. Adverse effects, including synechiae formation (*P* = 0.7639), edema (*P* = 0.7480), and infection (*P* = 0.5533) were not significantly different from the untreated side [51].

Other biomaterial agents such as oxidized methylcellulose [52], fibrin glue [53], microfibrillar collagen [44], and gelatin sponges [54] can be used to control intraoperative bleeding (e.g., cavernous bleeding). However, there is a lack of scientific evidence comparing the efficacy of these agents.

### 4.4. Surgical Techniques

#### 4.4.1. Hot Water Irrigation

Hot water irrigation was originally introduced as a treatment of epistaxis [55]. The hemostatic mechanism of hot water irrigation is unclear but may include (1) edema and narrowing of the intranasal lumen that contributes to the compression of the leaking vessel; (2) decreasing the flow and the intraluminal blood pressure due to mucosal vasodilatation; and (3) cleaning of blood coagulates from the nose [56]. Hot water irrigation for epistaxis is simple and effective, less painful, and less traumatic to the nose than nasal packing [57]; therefore, this technique was adopted to reduce intraoperative bleeding. Hot water irrigation with 40°–42° saline reduces diffuse oozing from sinonasal mucosa as well as intracranial bleeding from minor vessels [58]. Another benefit of warm water irrigation is that it allows the cleaning of the endoscopic lens. 

#### 4.4.2. Direct Control of Feeding Vessels

Sinonasal and skull base tumors, especially vascular tumors, are challenging for endoscopic skull base surgeons. One principle for the resection of vascular tumors is that of early control of the feeding vessels [58, 59], aiming for devascularization prior to extirpation. Moreover, having an effective instrument for coagulation, such as endoscopic bipolar electrocautery or radiofrequency coagulator, is important. Several studies have reported that coblation-assisted endoscopic resection of juvenile nasopharyngeal angiofibroma is associated with minimal morbidity and low-intraoperative blood loss [60, 61].

#### 4.4.3. Other

Visualization of the surgical field (making the anatomy more discernible) is greatly enhanced by preventing bleeding. Gentle manipulation is required for resection of tumors, especially those that are intradural, where the technique includes sequential subcapsular debulking, extracapsular sharp dissection, and gradual countertraction using meticulous and light suctioning. Avulsion of tumor tissue should be avoided as it may injure adjacent vital structures. In addition, adequate visualization of dissection planes, avoiding blind instrumentation, and avoiding direct or indirect injury from powered instruments (e.g., drills, microdebriders, and ultrasonic aspirators) are fundamental [38]. These techniques yield excellent control of the surgical field and a fluid surgery.

## 5. Conclusion

Numerous strategies are available to minimize intraoperative bleeding and improve the endoscopic surgical field. Regardless, anesthetic techniques (controlled hypotension, anesthetic agents) and surgical techniques (surgical techniques, topical vasoconstrictor, and hemostatic biomaterials) enhance the ability of each other to control bleeding. Therefore, cooperation between the anesthetic and surgical teams is of utmost importance to apply the appropriate techniques on each patient.

## Figures and Tables

**Table 1:** Various causes of bleeding tendency.

Components	Causes
Vascular	Inherited Disorder
	(i) Hereditary hemorrhagic telangiectasia
	(ii) Ehlers-Danlos syndrome
	Autoimmune disorder
	Allergic purpura
	Reduce the integrity of the blood vessel wall
	(i) Advanced age
	(ii) Prolonged steroid use
	(iii) Vitamin C deficiency

Platelets	Chronic diseases
	(i) Kidney failure
	(ii) Liver disease: hepatitis, cirrhosis, and liver failure
	(iii) Splenic sequestration
	(iv) Hematologic malignancy: leukemia, lymphoma, and multiple myeloma
	(v) Bone marrow diseases
	(vi) Human immunodeficiency virus/acquired immunodeficiency syndrome
	(vii) Rare autosomal recessive disorders (Glanzmann's thrombasthenia and Bernard-Soulier syndrome)
	Autoimmune diseases
	(i) Idiopathic thrombocytopenic purpura
	(ii) Systemic lupus erythematosus
	Medications
	(i) Antiplatelet: aspirin, nonsteroidal anti-inflammatory drugs (NSAIDs)
	(ii) Antibiotic including penicillin, quinine, and sulfa
	Dietary supplements
	Vitamin E, fish oil, *Echinacea, Aloe, Ginkgo*, Ginseng, and garlic extract

Coagulation factors	Inherited disorder
	(i) von Willebrand's disease
	(ii) Hemophilia
	(iii) Other inherited clotting factor deficiencies (factors II, V, VII, X, and XII)
	Medications
	(i) Warfarin (coumadin), heparin
	(ii) Chemotherapies
	(iii) Vitamin K deficiency
	Other disorders
	(i) Autoimmune disorders
	(ii) Disseminated intravascular coagulation (also results in thrombocytopenia)
	(iii) Liver disease

**Table 2:** Comparison of clinical trials study between inhalation-and intravenous-based techniques in controlled hypotension during endoscopic endonasal surgery.

Author (year)	Technique		Mean arterial pressure	Heart rate (beat/min)	Intraoperative bleeding	Surgical field score	Complication	Comment
Ankichetty et al. [23] (2011)	Inhalation based:	Iso/Fen	Mean time to achieve target MAP: 18 ± 8 min	No significant difference	No significant difference	No significant difference (*P* = 0.34)	No intra- and postoperative complications	No significant difference in sedation score, pain, nausea, vomiting, and hospital stay
	Intravenous based:	Pro/Fen	Mean time to achieve target MAP: 16 ± 7 min					

Yoo et al. [24] (2010)	Inhalation based:	Sevo/Rem	Initial: 91.1 ± 12.9 30 min: 67.1 ± 5.7 60 min: 67.2 ± 4.8	88.0 ± 21.8 77.1 ± 10.8 74.6 ± 10.7	N/A	2.21 (1–3)	N/A	No significant differences of MAP (*P* > 0.05), HR (*P* > 0.05), and surgical grade score (*P* = 0.83) among three groups
		Des/Rem	Initial: 92.2 ± 11.8 30 min: 67.4 ± 6.0 60 min: 67.3 ± 4.6	94.4 ± 20.0 78.2 ± 10.1 73.9 ± 12.1	N/A	2.07 (1–3)	N/A	
	Intravenous based:	Pro/Rem	Initial: 91.3 ± 11.3 30 min: 69.3 ± 6.7 60 min: 69.2 ± 5.2	79.1 ± 13.2 77.1 ± 15.7 74.9 ± 11.8	N/A	2.06 (1–3)	N/A	

Ragab and Hassanin [25] (2010)	Inhalation based:	Induction: Pro/Fen Maintenance: Iso/Fen/Emo	Mean time to achieve target MAP: 3 ± 1.4 min	Decreased by 20% ± 5%	Mean 120 ± 43 mL	VAS 4.2 ± 1.4 Six-point scale 1.6 ± 0.6	No serious adverse effects in both groups	(i) Significant difference in time to achieve target MAP and decreased HR (*P* < 0.05) (ii) Better visualization and bloodless in intravenous-based group (VAS; *P* = 0.005; Six-point scale; *P* = 0.02)
	Intravenous based:	Induction: Pro Maintenance: Pro/Rem	Mean time to achieve target MAP: 4 ± 1 min	Decreased by 24% ± 5%	Mean 100 ± 39 mL	VAS 3 ± 1.2 Six-point scale 1.2 ± 0.4		

Ahn et al. [26] (2008)	Inhalation based:	Sevo/Rem	N/A	N/A	135 mL/h (in patient with high LM score)	Numeric rating scales: 5.8 ± 2.3 (in patient with high LM score)	N/A	Significant better visualization in the high LM score patients in the intravenous based-group (blood loss *P* < 0.01; numeric rating scale *P* < 0.05); however, no significant difference in patient low LM score
	Intravenous based:	Pro/Rem	N/A	N/A	19 mL/h (in patient with high LM score)	Numeric rating scales: 2.3 ± 1.0 (in patient with high LM score)	N/A	

Beule et al. [27] (2007)	Inhalation based:	Sevo/Fen	70 ± 9.3	64.6 ± 6.6	Mean 4.1 ± 2.1 mL/min VAS of bleeding impending surgical progress 3.7 (1.9–8.4)	VAS 4.6 (3.0–7.8)	More impairment of platelet function in intravenous-based group (*P* = 0.0006)	No significant difference in all parameters (*P* > 0.05) Although test of platelet function showed more impairment in intravenous-based group, but it is not relevant to clinical practice
	Intravenous based:	Pro/Fen	71.5 ± 8.63	64.6 ± 7.3	Mean 3.8 ± 1.6 mL/min VAS of bleeding impending surgical progress 4.3 (2.4–7.8)	VAS 4.9 (3.9−7.5)		

Wormald et al. [28] (2005)	Inhalation based:	Sevo/Fen	71.65 ± 1.62	57.81 ± 1.69	N/A	2.8 ± 0.12	N/A	No significant differences in HR (*P* = 0.05), but significant difference in MAP (*P* = 0.045) and better surgical grade score in intravenous-based group (*P* < 0.001)
	Intravenous based:	Pro/Rem	67.43 ± 1.62	62.34 ± 1.69	N/A	2.21 ± 0.11	N/A	

Sivaci et al. [29] (2004)	Inhalation based:	Induction: Pro Maintenance: Sevo/Fen	Initial: 83 ± 5.7 15 min: 83 ± 3.7 30 min: 79 ± 4.0 45 min: 82 ± 6.7 60 min: 80 ± 6.3 End: 80 ± 4.7	76 ± 5.3 79 ± 4.3 71 ± 3.3 78 ± 2.7 79 ± 5.0 73 ± 5.3	Mean 296.9 ± 97.8 mL	N/A	N/A	(i) Significant lower blood loss in intravenous-based group (*P* < 0.01) (ii) No significant difference in MAP and HR (*P* > 0.05)
	Intravenous based:	Induction: Pro Maintenance: Pro/Fen	Initial: 81 ± 3.7 15 min: 84 ± 5.7 30 min: 82 ± 4.7 45 min: 83 ± 5.7 60 min: 84 ± 8.7 End: 85 ± 4.7	65 ± 3.7 66 ± 3.3 67 ± 4.1 75 ± 3.3 76 ± 4.0 72 ± 3.3	Mean 128.1 ± 37.3 mL	N/A	N/A	

Eberhart et al. [30] (2003)	Inhalation based:	Iso/Alf	67 (63–72) mmHg	72 (66–83)	Estimated blood loss 170 (100–270) mL	VAS 4.9 (3.5–4.7) Six-point scale 2 (2–4)	N/A	Significant decreased HR and better visualization in the intravenous based group (*P* ≤ 0.001); however, these results should be carefully interpreted due to using different opioids.
	Intravenous based:	Pro/Rem	65 (61–69) mmHg	55 (51–64)	Estimated blood loss 100 (50–240) mL	VAS 2.8 (2.0–3.4) Six-point scale 1 (1-2)	N/A	

Values are presented as mean ± SD or mean/medians (range); Iso: isoflurane; Sevo: sevoflurane; Des: desflurane; Pro: propofol; Fen: fentanyl; Rem: remifentanil; Esm: esmolol; Alf: alfentanil; VAS: visual analogue score; MAP: mean arterial pressure; HR: heart rate; LM: Lund-Mackay score; N/A: not available; *P: P*value*.*

**Table 3:** Review of controlled hypotension series in endoscopic endonasal surgery.

Author (Year)	Target mean arterial pressure	Grading quality of surgical field		Blood loss	Comment
Boezaart et al. [32] (1995)	>65 mmHg 60–64 mmHg 55–59 mmHg 50–54 mmHg	In SNP group* 3.63 ± 0.22 3.50 ± 0.50 3.31 ± 0.56 3.05 ± 0.55	In Esm group* 2.94 ± 0.34 2.82 ± 0.65 2.87 ± 0.39 2.48 ± 0.36	N/A	The optimum surgical conditions were provided with minimum Esm-induced hypotension (MAP > 65 mmHg); conversely, SNP induced hypotension (MAP 50–54 mmHg)

Jacobi et al. [33] (2000)	Moderate controlled hypotension with SNP (65–75 mmHg)	2.38 ± 0.77^§^		278 ± 110 mL	(i) Total blood loss and grading quality of surgical field did not show significant difference between the groups (ii) There was no correlation between blood loss and MAP
	Normotensive situation	2.25 ± 0.63^§^		245 ± 132 mL

Mengistu et al. [34] (2007)	Controlled hypotension group 50–55 mmHg	N/A		In Esm group 549 ± 208 mL In SNP group 557 ± 192 mL	Blood loss shows significant difference between the controlled hypotension group and controlled group
	Controlled group 70–80 mmHg	N/A		883 ± 465 mL

*Using quality scale is proposed by Fromme et al. [35];  ^§^Using numeric rating scale.

SNP: sodium nitropusside; Esm: esmolol; MAP: mean arterial pressure.

**Table 4:** Controlled bleeding techniques in endoscopic endonasal surgery.

Situation	Source	Technique
Artery		
Low-flow bleeding	Small perforating vessels Small arteriole	Bipolar electrocautery
		Hemostatic biomaterial agents
High-flow bleeding	Medium to large artery	Bipolar electrocautery
		Clips
		Angiography embolization

Venous		
Low-flow bleeding	Bleeding from mucosa, bone	Warm saline irrigation Drilling with diamond bur or applied bone wax with pledget (if presented focal bleeding from bone)
	Focal bleeding from venous sinus	Hemostatic biomaterial agents
High-flow bleeding	Venous sinus bleeding	Hemostatic biomaterial agents

## Abbreviations

HFJV: High-frequency jet ventilation
LMA: Laryngeal mask airway
NSAIDs: Nonsteroidal anti-inflammatory drugs
INR: International normalized ratio
LMWH: Low-molecular-weight heparin
n-BCA: n-Butylcyanoacrylate
EVOH: Ethyl-vinyl alcohol copolymer
ETCO_2_: End tidal carbon dioxide
IPPV: Intermittent positive pressure ventilation
GABA: Gamma-aminobutyric acid
NMDA: N-methyl d-aspartate
ACE: Angiotensin-converting enzyme
TIVA: Total intravenous anesthesia
GFR: Glomerular filtration rate
MAP: Mean arterial pressure
MAC: Minimum alveolar concentration
cGMP: Cyclic guanosine monophosphate.
